# *Tsukamurella tyrosinosolvens* (tsū-kə-mə-rel′lə tī′-rǝ-sē-nō-sol′vins)

**DOI:** 10.3201/eid3103.242004

**Published:** 2025-03

**Authors:** Clyde Partin

**Affiliations:** Emory University School of Medicine, Atlanta, Georgia, USA

**Keywords:** Tsukamurella tyrosinosolvens, etymology, bacteria

The species name for the bacterium *Tsukamurella* *tyrosinosolvens* was accepted in 1997 on the basis of biochemical attributes: *tyrosina*, from the amino acid tyrosine in cheese (*tyros*, Greek for cheese), which imparts a crystalline texture (Figure). The hydrolysis, or dissolving of tyrosine—thus, *tyrosinosolvens*—is a species characteristic. 

**Figure:**
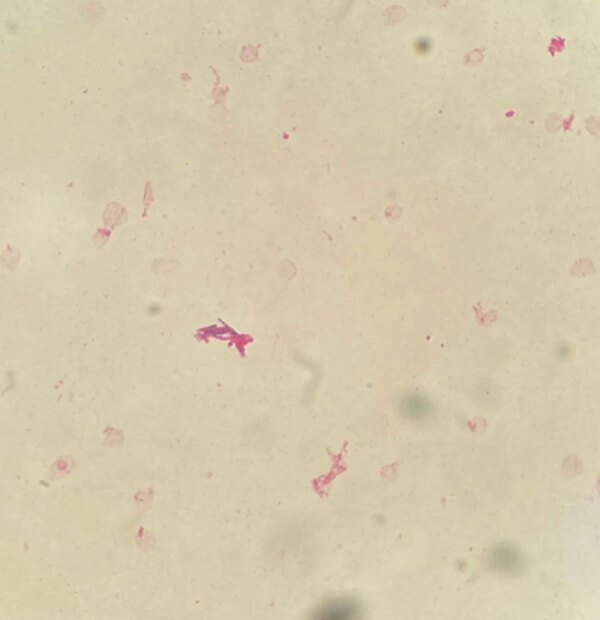
Gram staining from aerobic blood cultures (×1,000 magnification) showing numerous long, slightly curved, thin, nonbranching, and gram-positive rods, confirmed as *Tsukamurella tyrosinosolvens*. Image from (*2*); licensed by CC by 4.0 (https://creativecommons.org/licenses/by/4.0).

The genus *Tsukamurella* consists of commensal bacteria with a propensity to cause opportunistic infections in immunocompromised patients, especially those with chronic lung disease. *Tsukamurella* bacteria are related to the genera *Nocardia*, *Mycobacterium*, *Corynebacterium*, and *Gordonia. Gordonia aurantiaca* was initially isolated in 1971 by renowned Japanese physician-microbiologist and *Mycobacteria* taxonomist Michio Tsukamura at Nagoya University in Nagoya, Japan. In 1988, he was honored with the genus name *Tsukamurella*. 

*Tsukamurella*, retrospectively isolated by Edward A. Steinhaus in 1941 from the mycetoma and ovaries of the bedbug, was originally misidentified as *Corynebacterium paurometabolum*. *Tsukamurella* are weakly acid-fast; therefore, clinical manifestations can be confused with those of tuberculosis and create microbiological misidentification with *Mycobacterium* and *Corynebacterium* spp.

*Tsukamurella* infections are rare and usually associated with immune-suppressed patients, but severe infections have occurred in immunocompetent patients. Currently, of 17 known species, 12 cause human disease. Although *Tsukamurella* infections have been increasingly reported in Europe, Asia, America, and Africa, *T. tyrosinosolvens* has been the most common species observed.
